# Optimizing predictions in IRRI’s rice drought breeding program by leveraging 17 years of historical data and pedigree information

**DOI:** 10.3389/fpls.2022.983818

**Published:** 2022-09-20

**Authors:** Apurva Khanna, Mahender Anumalla, Margaret Catolos, Sankalp Bhosale, Diego Jarquin, Waseem Hussain

**Affiliations:** ^1^Rice Breeding Innovation Platform, International Rice Research Institute (IRRI), Los Baños, Laguna, Philippines; ^2^Agronomy Department, University of Florida, Gainesville, FL, United States

**Keywords:** rice, drought, pedigree-based predictions, G × E interactions, general combining ability, specific combining ability

## Abstract

Prediction models based on pedigree and/or molecular marker information are now an inextricable part of the crop breeding programs and have led to increased genetic gains in many crops. Optimization of IRRI’s rice drought breeding program is crucial for better implementation of selections based on predictions. Historical datasets with precise and robust pedigree information have been a great resource to help optimize the prediction models in the breeding programs. Here, we leveraged 17 years of historical drought data along with the pedigree information to predict the new lines or environments and dissect the G × E interactions. Seven models ranging from basic to proposed higher advanced models incorporating interactions, and genotypic specific effects were used. These models were tested with three cross-validation schemes (CV1, CV2, and CV0) to assess the predictive ability of tested and untested lines in already observed environments and tested lines in novel or new environments. In general, the highest prediction abilities were obtained when the model accounting interactions between pedigrees (additive) and environment were included. The CV0 scheme (predicting unobserved or novel environments) reveals very low predictive abilities among the three schemes. CV1 and CV2 schemes that borrow information from the target and correlated environments have much higher predictive abilities. Further, predictive ability was lower when predicting lines in non-stress conditions using drought data as training set and/or *vice-versa*. When predicting the lines using the data sets under the same conditions (stress or non-stress data sets), much better prediction accuracy was obtained. These results provide conclusive evidence that modeling G × E interactions are important in predictions. Thus, considering G × E interactions would help to build enhanced genomic or pedigree-based prediction models in the rice breeding program. Further, it is crucial to borrow the correlated information from other environments to improve prediction accuracy.

## Introduction

Rice (*Oryza sativa* L.) is one of the important cereal crops globally, providing food to more than 3.5 billion people. The key goal of rice breeding across the globe is to develop high-yielding rice varieties to meet the rice food demands (Xu et al., 2021). Conventional breeding coupled with marker-based selection has been successful and led to positive increase in rice productivity. However, the rate of genetic improvement in rice has not been at a pace to meet the expected rice food demands of 1.5% or above for the growing population (Khanna et al., 2022). For example, the rate of genetic gain in the International Rice Research Institute’s (IRRI) global rice breeding program is <1% (Juma et al., 2021; Kumar et al., 2021; Khanna et al., 2022), which is not sufficient to meet future rice demands. Thus, it is necessary to increase the rate of genetic gain in rice to ensure future food security (Peng et al., 2004; Li et al., 2018). One strategy to increase the rate of genetic gain in rice breeding programs is to complement conventional rice breeding with modern tools and technologies. One such tool is the genomic selection which can increase rates of genetic gain by reducing the breeding cycle duration or by increasing the selection accuracy (Meuwissen et al., 2001). In genomic selection, the predicted phenotypic values can be used as alternatives to phenotypic values observed in field experiments, which may help to accelerate breeding programs by skipping field experiments for selections. Thus, it is expected to increase selection gains per unit time (Krishnappa et al., 2021).

In prediction assisted selection, genetic relationships are either based on pedigrees or molecular markers or both to predict untested genotypes’ performance. As compared to pedigrees, more recently, molecular markers have been found to significantly increase the prediction accuracy of genomic estimated breeding values (GEBVs), as molecular markers account for the genetic variations due to the Mendelian sampling (Crossa et al., 2010; Albrecht et al., 2011; Burgueño et al., 2012; Sukumaran et al., 2017; Velazco et al., 2019; Krishnappa et al., 2021). Compared to the estimation of GEBVs, pedigree-based breeding values of un-phenotyped lines account for mid-parent genetic contributions and not for Mendelian segregation of alleles, which are more or less expected due to random chance. However, researchers have found conclusive evidence that, in some instances, pedigree-based relationships may perform similarly to or even better than marker-based relationships in terms of prediction accuracy and estimating breeding values (Rutkoski et al., 2016; Juliana et al., 2017; Hunt et al., 2018). This is particularly true when the pedigree data are precise and include several generations (Juliana et al., 2017). Further, the implementation of pedigree-based predictions (PBP) without accounting for the genotype-by-environment G × E interaction *via* A × E effects may be trivial in crop breeding as yield, and yield-related traits are strongly influenced by environmental factors. PBP accounting for by A × E interaction effects will be beneficial and more accurate to predict the performance of untested genotypes under a targeted environment and, finally, lead to higher genetic gains per cycle (Burgueño et al., 2012; Jarquín et al., 2014; Pérez-Rodríguez et al., 2015; Sukumaran et al., 2017; Ovenden et al., 2018; Jarquin et al., 2021; Rogers et al., 2021; Rogers and Holland, 2022).

Several crop researchers have been very keen on using genomic selection (GS) and dissecting G × E by leveraging historical data to help build prediction models and to improve the current selection breeding programs (Dawson et al., 2013; Lado et al., 2016; Dreisigacker et al., 2021; Rogers and Holland, 2022). In rice, assessing the prediction accuracy including the G × E interaction has not been considered yet, particularly with a large number of environmental data sets. Further, the appropriate strategy for predicting trait performance of genotypes in challenging prediction scenarios (novel environments) has not been considered yet in rice breeding. Utilizing huge historical data sets spanning over many environments can help us to enhance the predictive ability of models by leveraging the G × E interaction. The best strategy to predict trait performance of genotypes in unobserved environments in rice is still unclear. Here, we leverage 17 years of historical data with robust pedigree information evaluated under non-stress (normal) and reproductive stage drought stress conditions at IRRI. The main objective of the study was to utilize the large historical dataset for model calibration, and incorporate it in improved models to: (a) assess the prediction performance of lines under different cross-validation schemes of interest for breeders, (b) compare the predictive ability of various models when predicting phenotypic responses in already observed and novel or new environments, also varying the training set composition with genotypes tested in similar (stress to predict stress; non-stress to predict non-stress), combined (stress and non-stress to predict stress, stress and non-stress to predict stress), and different (stress to predict non-stress, non-stress to predict stress) conditions, and (c) dissect the genotype × environment interaction, through the pedigree × environment component.

## Materials and methods

### Description of historical phenotypic data

The study utilized the historical data from the breeding trials conducted under control (non-stress) and reproductive stage drought stress conditions from 2003 to 2019 at IRRI, Philippines. The trials were conducted twice a year in consecutive, dry (from January to April) and wet (from late June to September) seasons. The dataset is an aggregation of 53 trials, with 19,826 data points. Total number of unique genotypes evaluated was 2,490, with 33 checks across all the trials. Further, seven checks were frequently used in most of the trials thereby connecting the dataset across years. The breeding trials were organized in varying designs including alpha-lattice, augmented randomized complete block, and randomized complete block designs (RCBD). Three major agronomic traits days to 50% flowering (DTF), plant height (PH), and grain yield (kg/ha) were retrieved and used for downstream analysis.

### Pedigree data extraction

The pedigree data of 2,490 unique genotypes were retrieved from IRRIs “Breeding 4 Results” database (Breeding 4 Results (B4R), 2021, https://b4r.irri.org). The pedigree data of immediate parents as well as grandparents upto seven generations was retrieved (Hussain et al., 2022; Khanna et al., 2022). The number of parental lines was reduced from 2,490 lines to 361 lines by the seventh generation. Additionally, the cross-type information was extracted from IRRI’s genealogy management system using the standard R pedigree retrieval pipeline (McLaren et al., 2005; Collard et al., 2019). The R package AGHMatrix was used for constructing the pedigree A-matrix (Amadeu et al., 2016).

### Statistical analysis

#### Pre-processing and quality check of data

Before the statistical modeling, the data was pre-processed, and the quality of phenotype data was thoroughly checked to ensure only high-quality data trials and phenotypes were forwarded for downstream analysis. The pre-processing and quality procedure followed is thoroughly described in Khanna et al. (2022). Briefly, the trials with lack of proper experimental design, replications, and having more than 20% of missing data for grain yield were discarded. Phenotypic data was also checked for extreme values and outliers using the Bonferroni-Holm test for studentized residuals test (Bernal-Vasquez et al., 2016; Philipp et al., 2019). After quality check, 53 trials harboring 19,828 phenotypic data points with 2,490 unique lines were retained for phenotypic data analysis for estimating the breeding values.

#### Statistical modeling of phenotypic data

For the analysis, a two-stage approach of mixed-model was used to analyze the data for grain yield, DTF, and PH (Piepho et al., 2008, 2012; Smith and Cullis, 2018) under non-stress, drought, and by combined analyses of drought and non-stress together. The two-stage approach was adopted to account for different experimental designs across the environments (Damesa et al., 2017).

In the first stage, BLUEs per environment for each genotype were estimated for the drought and non-stress trials separately. The mixed model utilized is as follows:


(1) $$y_{\mathrm{ijkl}}=\mu +g_{i}+r_{j}+b_{k}+s_{l}+\varepsilon_{\mathrm{ijkl}}$$

where, $y_{\mathrm{ijkl}}$ represents adjusted mean for *i*th observation in *j*th replication, *k*th block and *l*th season, *μ* is the overall mean, *g_i_* is the fixed effect of *i*th genotype, *r_j_* is the random effect of replications in each trial, *b_k_* is the random block effect, *s_l_* is the random effect for season and $\varepsilon_{\mathrm{ijkl}}$ is the residual error. Random effects were assumed to be independently and identically distributed following normal densities. In the above model, DTF was used as a covariate for reducing the error on yield caused due to the presence of different maturity genotypes. Further, the above model was used for the trials which were performed using an alpha-lattice breeding design.

Regarding the combined analysis, a linear mixed model was used to extract a single value for each genotype (BLUE) across the non-stress and drought treatments. The model utilized is as follows:


(2) $$y_{\mathrm{ijklm}}=g_{i}+r_{j}+b_{k}+s_{l}+t_{m}+\varepsilon_{\mathrm{ijklm}}$$

all the terms are similar to those described in Equation (1), except the $t_{m}$ which is the fixed effect of *m*th treatment (either non-stress or reproductive stage drought stress). In the above model different variances across non-stress and drought treatments were assumed.

Heritabilities under non-stress and drought for grain yield were calculated as per Cullis et al., 2006 and Piepho and Möhring, 2007. Heritability was calculated as follows:


(3) $$H^{2}=1-\frac{{\bar{V}}_{\mathrm{BLUP}}}{2\sigma_{g}^{2}}$$

where, ${\bar{V}}_{\mathrm{BLUP}}$is the mean–variance difference of two BLUPs and *σ*^2^*g* is the variance of genotypes.

In the second stage analysis, a pedigree-based mixed model approach was used to perform predictions in non-stress, drought, and combined data. The pedigree-based models (Models 1 and 2) for main additive effects and the inclusion of interaction with environments are described. Then the models were extended to incorporate the general combining ability (GCA) and specific combining ability (SCA) terms (Models 3–7). The purpose to introduce GCA and SCA based models in this study was to leverage the phenotypic information of the parents involved in more than one cross combination (Jarquin et al., 2021). For instance, if we consider a hypothetical cross P_W × P_Z derived from the cross between parent 1 (P_W) and parent 2 (P_Z), it might be the case that parent P_W was also partially observed in other hypothetical crosses P_W × P_A, P_D × P_W, P_W × P_H, and P_N × P_W while parent P_Z in crosses P_B × P_Z, P_J × P_Z, and P_Z × P_S. In these cases, both parents were part of other cross combinations for which there is also phenotypic data. Thus, for leveraging the phenotypic information of the parents involved in other crosses, we proposed a set of models based on pedigree data that also allow the inclusion of the GCA and SCA terms. In this section, we describe these models.

#### Model 1: E + A

Suppose that the performance of the *i*th (*i* = 1, 2, …, *L*) genotype in the *j*th (*j* = 1, 2, …, *J*) environment $\left(y_{ij}\right)$ can be explained by a model that accounts for the main effect of the environments $\left(E_{j}\right)$, the additive effect of the genotypes based on pedigree information $\left(a_{i}\right)$ and an error term capturing the non explained variability $\left(\varepsilon_{ij}\right)$ as follows.


(4) $$y_{ij}=E_{j}+a_{i}+\varepsilon_{ij}$$

where $E_{j}$ is assumed to follow identically and independent distributed (IID) normal densities such that $E_{j}\sim N\left(0,\sigma_{E}^{2}\right)$, $\sigma_{E}^{2}$ is the corresponding variance component; the vector of additive effects $a=\left\{a_{i}\right\}$ follows a multivariate normal density with mean on zero and a co-variance matrix $cov\left(a\right)=A\sigma_{a}^{2}$, where ***A*** is the pedigree matrix and its entries describe genetic similarities between pairs of lines, and $\sigma_{a}^{2}$ is the corresponding variance component such that $a\sim N\left(0,A\cdot \sigma_{a}^{2}\right)$, and $\varepsilon_{ij}\sim N\left(0,\sigma^{2}\right)$.

#### Model 2: E + A + A × E

Model 2 is an extension of Model 1 that includes the interaction between genotypes based on pedigree information and environments.


(5) $$y_{ij}=E_{j}+a_{i}+aE_{ij}+\varepsilon_{ij}$$

The interaction term was included by considering the Hadamard product between two co-variance structures (Jarquín et al., 2014; Pérez-Rodríguez et al., 2015). Suppose that $aE_{ij}$ represent the interaction between the *i*th genotype and the *j*th environment such that the vector of interactions $aE=\left\{aE_{ij}\right\}\sim N\left(0,Z_{g}AZ_{g}^{\prime }\#Z_{E}Z_{E}^{\prime }\cdot \sigma_{aE}^{2}\right)$ and with $\sigma_{aE}^{2}$ as the corresponding variance component, and $Z_{g,}Z_{E}$ are the incidence matrices that connect phenotypes with lines and environments, respectively.

#### Model 3 (E + GCA): E + A_P1_ + A_P2_

This model decomposes the genetic effect as the sum of the paternal $a_{P1i}$ and maternal $a_{P2i}$ effects. In our case, we called these parents 1 and parent 2 only. The proposed linear predictor is as follows:


(6) $$y_{ij}=E_{j}+a_{P1i}+a_{P2i}+\varepsilon_{ij}$$

were $a_{P1}=\left\{a_{P1i}\right\}$ and $a_{P2}=\left\{a_{P2i}\right\}$ represent corresponding vectors of additive effects from parent 1 and parent 2 and these follow multivariate normal densities with mean on zero and co-variance matrices $cov\left(a_{P1}\right)=A_{P1}\cdot \sigma_{aP1}^{2}$and $cov\left(a_{P2}\right)=A_{P2}\cdot \sigma_{aP2}^{2}$, $A_{P1}$and $A_{P2}$are the pedigree matrices of the parents involved in the crosses, and $\sigma_{aP1}^{2}$ and $\sigma_{aP2}^{2}$ are the corresponding variance components such that $a_{P1}\sim N\left(0,\;A_{P1}\cdot \sigma_{aP1}^{2}\;\right)$ and $a_{P2}\sim N\left(0,\;A_{P2}\cdot \sigma_{aP2}^{2}\;\right)$.

#### Model 4 (E + GCA + SCA): E + A_P1_ + A_P2_ + A_P1×P2_

This model is an extension of model 3 that also includes the interaction effect of crossing a specific pair of parents $a_{P1\times P2\_i}$ (parent 1 and parent 2). The obtained linear predictor is as mentioned below


(7) $$y_{ij}=E_{j}+a_{P1i}+a_{P2i}+a_{P1\times P2\_i}+\varepsilon_{ij}$$

were, $a_{P1\times P2}=\left\{a_{P1\times P2\_i}\right\}$ represent the vector of interaction effects derived from crossing respective parents, parent 1 and parent 2, and it follows a multivariate normal density with mean on zero and a co-variance matrix $cov\left(a_{P1\times P2}\right)=Z_{P1}A_{P1}Z_{P1}^{\prime }\#Z_{P2}A_{P2}Z_{P2}^{\prime }\cdot \sigma_{aP1\times P2}^{2}$ such that $a_{P1\times P2}\sim N\left(0,Z_{P1}A_{P1}Z_{P1}^{\prime }\#Z_{P2}A_{P2}Z_{P2}^{\prime }\cdot \sigma_{aP1\times P2}^{2}\;\right)$ with $\sigma_{aP1\times P2}^{2}$ as the corresponding variance component, $Z_{P1}$ and $Z_{P2}$ are the corresponding incidence matrices that connect phenotypes with the respective parents of the cross (1 and 2), and “#” represents the cell-by-cell product between two matrices also known as the Hadamard product.

#### Model 5 (E + GCA + SCA + GCA×E + SCA×E): E + A_P1_ + A_P2_ + A_P1×P2_+ A_P1_ × E + A_P2_ × E + A_P1×P2_ × E

This model is an extension of model 4 that also includes the interaction between the GCA components and the environment, and between the SCA component and the environment. The obtained linear predictor is as follows:


(8) $$\begin{array}{c} y_{ij}=E_{j}+a_{P1i}+a_{P2i}+a_{P1\times P2\_i}\\ \;+aE_{P1ij}+aE_{P2ij}+aE_{P1\times P2\_ij}+\varepsilon_{ij} \end{array}$$

were $aE_{P1}=\left\{aE_{P1ij}\right\}$ and $aE_{P2}=\left\{aE_{P2ij}\right\}$represent the interaction effect vectors between parent 1 and the environment, and between parent 2 and the environment, and these follow multivariate normal densities with mean on zero and a co-variance matrices $cov\left(aE_{P1}\right)=Z_{P1}A_{P1}Z_{P1}^{\prime }\#Z_{E}Z_{E}^{\prime }\cdot \sigma_{aE\_P1}^{2}$ and $cov\left(aE_{P2}\right)=Z_{P2}A_{P2}Z_{P2}^{\prime }\#Z_{E}Z_{E}^{\prime }\cdot \sigma_{aE\_P2}^{2}$ such that $aE_{P1}\sim N\left(0,Z_{P1}A_{P1}Z_{P1}^{\prime }\#Z_{E}Z_{E}^{\prime }\cdot \sigma_{aE\_P1}^{2}\right)$ and $aE_{P2}\sim N\left(0,Z_{P2}A_{P2}Z_{P2}^{\prime }\#Z_{E}Z_{E}^{\prime }\cdot \sigma_{aE\_P2}^{2}\right)$, $\sigma_{aE\_P1}^{2}$ and $\sigma_{aE\_P2}^{2}$ are the associated variance components, $Z_{E}$ is the incidence matrix that connects phenotypes with environments; $aE_{P1\times P2}=\left\{aE_{P1\times P2\_ij}\right\}$ the interaction term between SCA term and environments follows a normal density such that $aE_{P1\times P2}\sim N\left(0,Z_{P1}A_{P1}Z_{P1}^{\prime }\#Z_{P2}A_{P2}Z_{P2}^{\prime }\#Z_{E}Z_{E}^{\prime }\sigma_{aE\_P1\times P2}^{2}\right)\text{,}$and $\sigma_{aE\_P1\times P2}^{2}$ represents the associated variance component.

#### Model 6 (E + GCA + SCA + GCA×E + A × E): E + A_P1_ + A_P2_ + A_P1×P2_+ A_P1_ × E + A_P2_ × E + A × E

Model 6 is a combination between models 2 and 6 where the SCA × E (***A***_P1×P2_ × ***E***) component was substituted by the interaction between genotypes and environments (***A*** × ***E***). The linear predictor is as follows:


(9) $$\begin{array}{c} y_{ij}=E_{j}+a_{P1i}+a_{P2i}+a_{P1\times P2\_i}\\ \;+aE_{P1ij}+aE_{P2ij}+aE_{ij}+\varepsilon_{ij} \end{array}$$

where, all of the terms remain as before defined.

#### Model 7 (E + GCA + A + GCA×E + A × E): E + A_P1_ + A_P2_ + A + A_P1_ × E + A_P2_ × E + A × E

Model 7 is also a combination between models, in this case between models 2 and 3, where the SCA (***A***_P1×P2_) and the SCA × E (***A***_P1×P2_ × ***E***) are replaced by the hybrid main effect based on pedigree data and the interaction between hybrids and environments (***A*** × ***E***). The linear predictor is as follows:


(10) $$y_{ij}=E_{j}+a_{P1i}+a_{P2i}+a_{i}+aE_{P1ij}+aE_{P2ij}+aE_{ij}+\varepsilon_{ij}$$

where, all of the terms remain as before defined.

#### Cross-validation schemes

For assessing the proficiency of the aforementioned seven regression models, three cross-validation scenarios CV2, CV1, and CV0 mimicking real prediction scenarios of interest for breeders at different stages of the breeding pipeline were considered in this study. A brief description of these scenarios is outlined below. For each cross-validation scheme, three data sets were considered and these correspond to lines tested under (i) drought, and (ii) non-stress conditions, and (iii) combined (non-stress and drought). In addition, a crossed validation was considered where the non-stress dataset was used to predict the grain yields under drought conditions and *vice-versa*. For all of the validation scenarios, no matter how the training sets were composed the correlation between predicted and observed values was computed within each environment-stress condition combination such that the results are comparable across data sets (drought stress, non-stress, and combined). In addition, extra care was taken to have same partitions in testing sets across cross-validation schemes for the different ways to compose training sets (only stress, only non-stress, and combined) when predicting crop performance in stress and non-stress conditions.

- **CV2:** Predicting the performance of incomplete trials, lines tested in some environments/years but not in others. A random five-fold cross-validation was followed wherein one-fold was used for validation and the remaining four folds were used for model training/calibration, i.e., performance of 20% of the lines in environments (phenotypes) were predicted using remaining 80% of the observed phenotypes. Here, the performance of 20% of the phenotypes is observed in few environments but not others. After integrating the predicted values into a single vector, the values derived from the five-fold cross-validation, the correlation between the predicted and the observed values was computed within environments and the weighted average correlation was reported as the prediction accuracy (Jarquín et al., 2014). In this study, 10 replicates of randomly assign phenotypes to folds were considered, and the mean weighted average prediction accuracy across the 10 replicates was reported in tables and plots.
- **CV1:** Predicting newly developed lines that have not been evaluated in any environment (Jarquín et al., 2017) yet. As in CV2, a five-fold cross-validation was considered with one-fold used for validation and the remaining four folds for training the model. However, in this case around 20% of the lines in the testing set are not observed in any of the environments. In this scenario, also 10 replicates for randomly assign genotypes to folds were considered and the mean weighted average correlation was reported as well.
- **CV0:** Predicting the performance of already observed genotypes but in a new/novel/unobserved environment/year (prediction leaving-one-year-out) where these have not been observed yet. The crop performance in a given year is predicted using the performance of lines from all the available years. To form the training set, the data from all the years are combined except for 1 year that is excluded, and the left-out year was used as a validation/testing set. For example, the data from 2003 to 2018 was combined to form the training set while the year 2019 was used as a validation set. Similarly, data from 2003 to 2017 and 2019 was used as training set while the year 2018 was used as a validation set, and so on.

## Results

The study was undertaken to assess the usefulness of using historical data for improving predictive ability of rice by considering a series of proposed models having interaction effects of their components with the environment. For this, three cross-validation scenarios (CV0, CV1, and CV2) were considered together with the crossed validation between groups of environmental stress management/water availability (stress, and non-stress). In this study, 17 years of historical data of IRRI’s drought breeding program including phenotypic and pedigree data were used for analysis. The purpose of using pedigree-based relationship matrix was to allow the borrowing of information between genotypes by accounting for the additive genetic covariance and the interaction with the environments for enhancing the precision of breeding value estimation (Piepho et al., 2008).

### Characteristic features of the data

**Figure 1:**
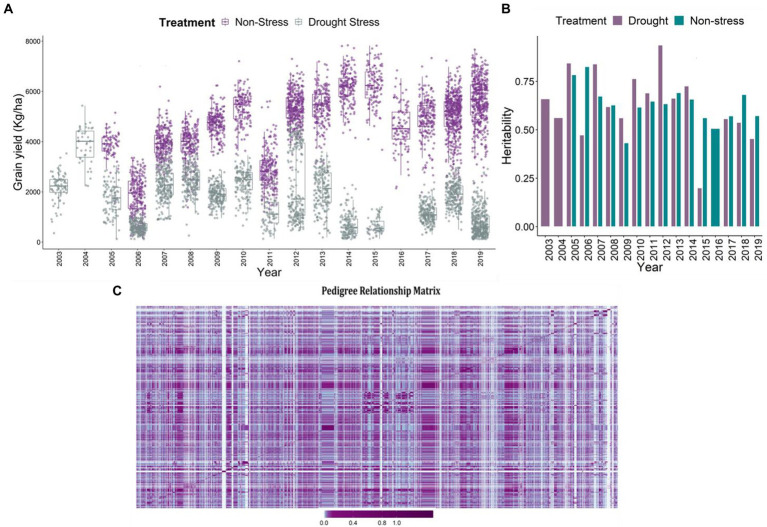
**(A)** Boxplot projecting adjusted mean values for the grain yield (kg/ha) under non-stress and reproductive stage drought stress conditions from the year 2003 to 2019. The *x*-axis depicts the years, which are considered as environments. The yield penalty owing to drought stress imposition led to the reduction of the grain yields of the genotypes. **(B)** The plot represents the heritability of the trials in each year from 2003 to 2019 under stress and non-stress conditions. The violet bars represent drought stress trials and cyan represents the non-stress trials. **(C)** Pedigree relationship matrix of the genotypes tested across years from 2003 to 2019. The connectivity of the genotypes is evident from the matrix, checks and reproductive stage drought tolerant varieties were tested in the successive years, thereby connecting the dataset across years. The violet color depicts the higher connectivity, marginalizing towards cyan depicting moderate to low relationship.

The major agronomic trait of interest was grain yield under reproductive stage drought stress, non-stress and combined conditions. The descriptive statistics of the data is detailed in Khanna et al. (2022). Huge difference in grain yield was observed in the grain yield (kg/ha), between the drought and non-stress conditions (Figure 1A). The adjusted means for grain yield varied between 184.75 and 7,834.19 kg/ha in non-stress conditions, and 18.26–5430.72 kg/ha under drought stress. Low grain yield observed under drought conditions indicates the impact of drought on the final grain yield. Heritability for grain yield across the drought stress trials ranged between 0.2 and 0.94 (Figure 1B). However, under the non-stress conditions it varied between 0.43 and 0.83. Among the total 17 trials, eight trials executed under reproductive stage drought stress conditions depicted extremely lower heritabilities in comparison to the non-stress trials. The reduced heritability values of the stress trials have been evident owing to compromised grain yields of the genotypes under reproductive stage drought stress conditions (Henry et al., 1997; Kumar et al., 2007).

### Pedigrees and data connectivity

The A-matrix was formulated using pedigree information of the parental lines upto seven generations to ensure appropriate pedigree-depths which would in turn help estimate true kinship coefficients (Amadeu et al., 2016). The pedigree matrix explaining the clustering and relatedness of 2,490 unique individuals is depicted in Figure 1C. In terms of data connectivity across years for reliable estimates or predictions, we observe good data connectivity of genotypes across the years. The good connectivity in the data was a consequence of having long-term checks (IR64, Swarna, Sahbhagi Dhan, IRRI 154) used across the years in the breeding program. Additionally, in the breeding program, the selected superior genotypes were forwarded and re-evaluated in the succeeding years establishing good connectivity in the data (Figure 1C). Further, to enhance the connectivity of the lines and have reliable estimates of the breeding values, the kinship matrix based on pedigrees of 2,490 unique lines was used in the second stage of the prediction analysis. The purpose of using the relationship matrix based on pedigrees was to connect the lines across years by borrowing information from parents and grandparents (Khanna et al., 2022).

### Predictions using three cross-validation schemes

**Figure 2:**
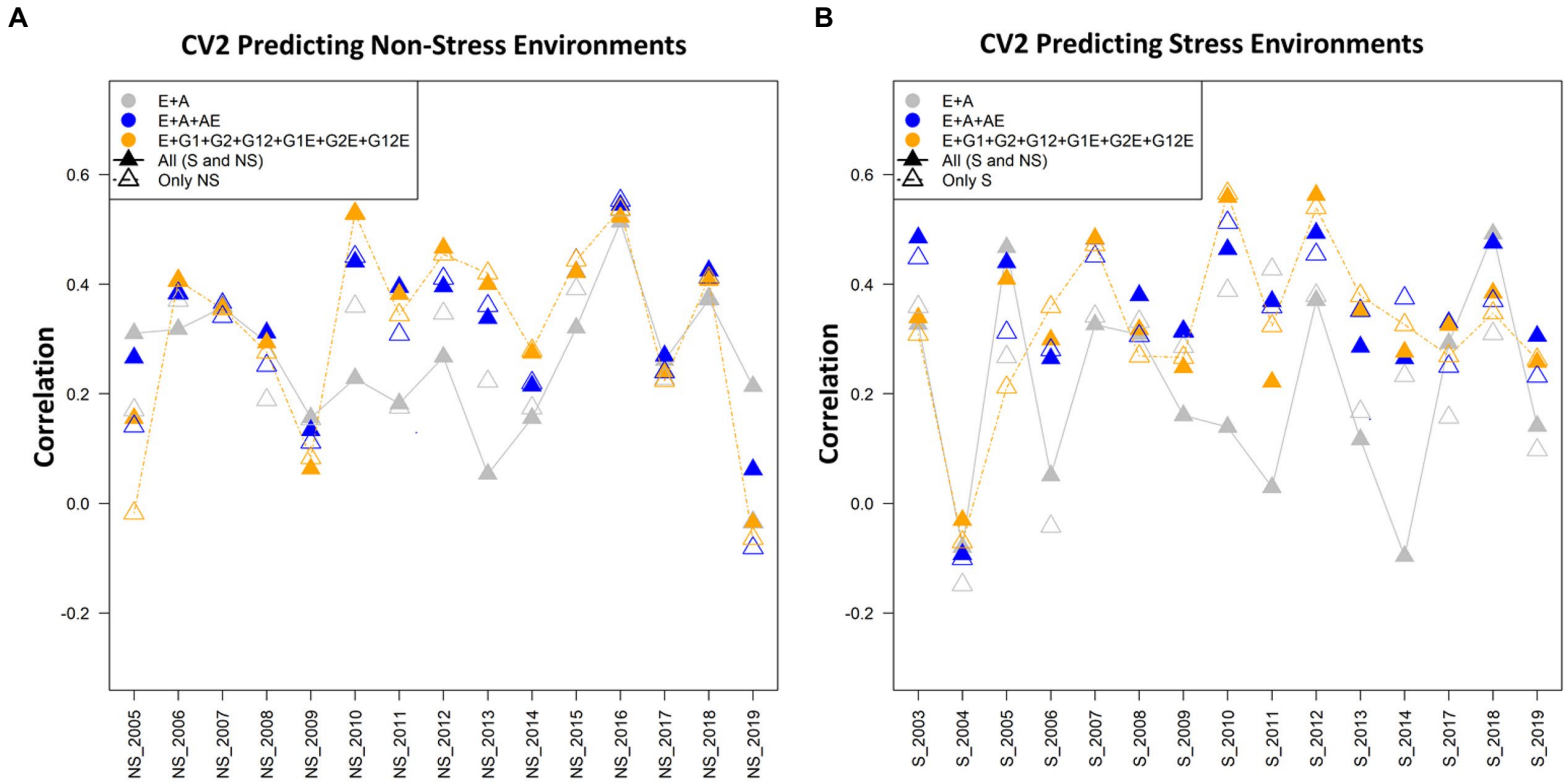
**(A)** The average correlation values obtained from predicting the grain yield for incomplete trials (CV2) for non-stress conditions with the non-stress (NS) and combined [All (S and NS)] calibration datasets. The plot depicts the correlations between the predicted and estimated values across the years 2005–2019, using model 1 (E + A), 2 and 5, respectively. **(B)** The average correlation values obtained from predicting the grain yield for incomplete trials (CV2) under the stress conditions using the stress (S) and combined [All (S and NS)] calibration datasets. The plot depicts the correlations between the predicted and estimated values across the years 2003–2019, using model 1 (E + A), 2 and 5, respectively.

**Figure 3:**
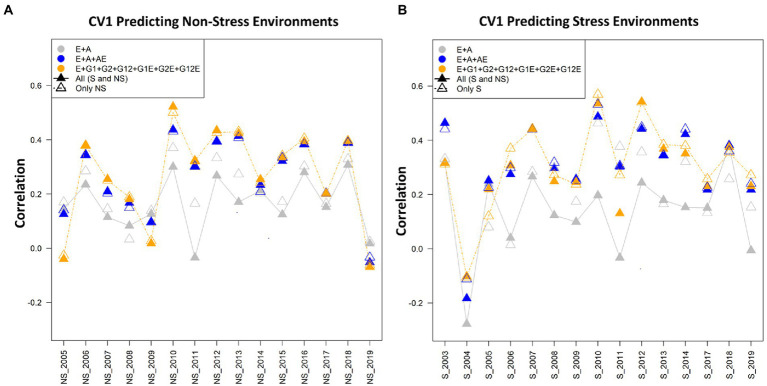
**(A)** The average correlation values obtained from predicting the grain yield for untested lines (CV1) for predicting non-stress conditions using the non-stress (NS) and combined [All (S and NS)] calibration sets. The plot depicts the correlations between the predicted and estimated values across the years 2005–2019, using model 1 (E + A), 2 and 5, respectively. **(B)** The average correlation values obtained from predicting the grain yield for untested lines (CV1) under the stress conditions using stress (S) and combined [All (S and NS)] datasets. The plot depicts the correlations between the predicted and estimated values across the years 2003–2019, using model 1 (E + A), 2 and 5, respectively.

**Figure 4:**
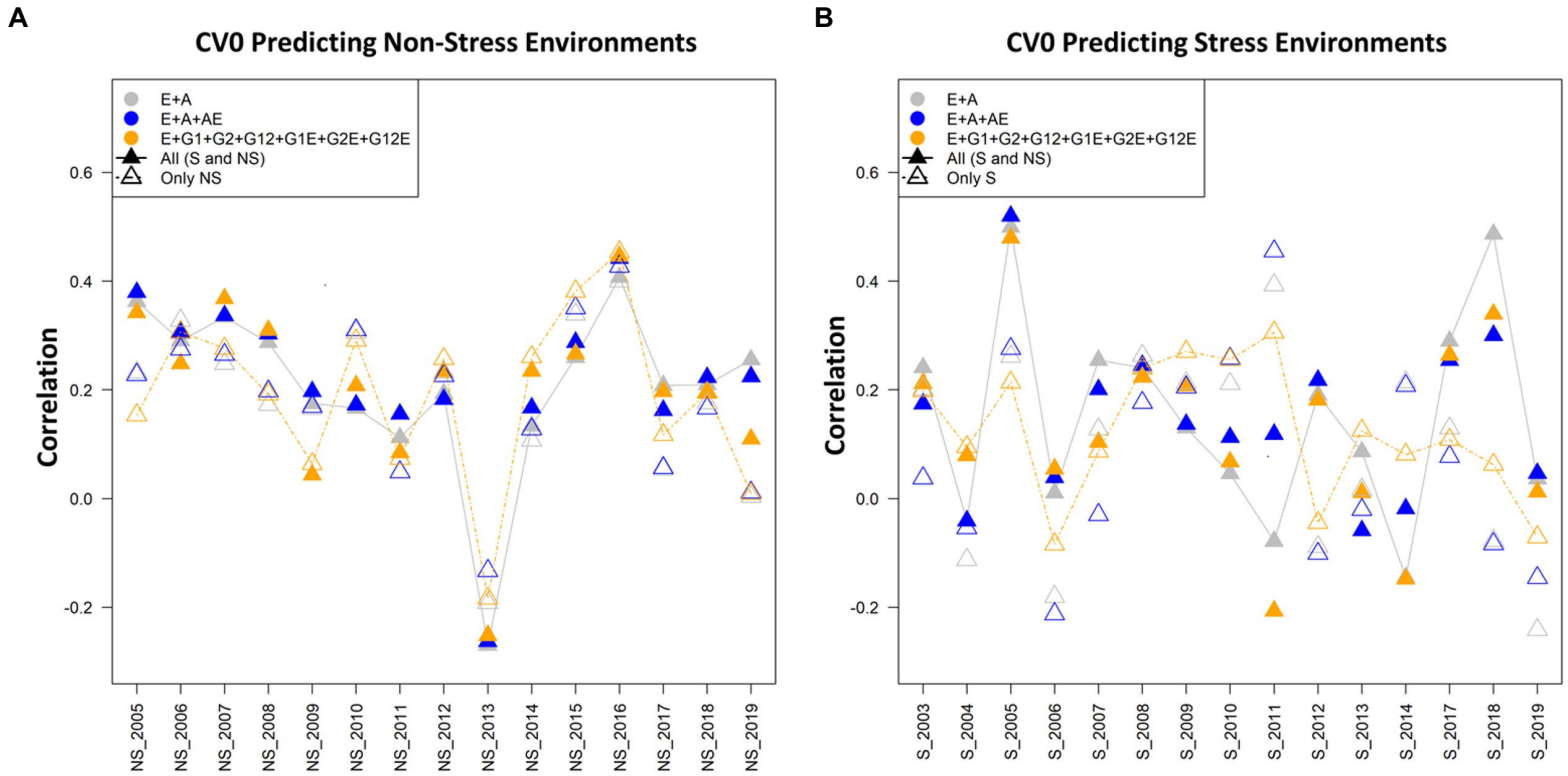
**(A)** The average correlation values obtained from predicting the grain yield for new environment/year (CV0) under the non-stress conditions using non-stress (NS) and combined [All (S and NS)] datasets. The plot depicts the correlations between the predicted and estimated values across the years 2005–2019, using model 1 (E + A), 2 and 5, respectively. **(B)** The average correlation values obtained from predicting the grain yield for new environment/year (CV0) under the stress conditions using the stress (S) and combined [All (S and NS)] datasets. The plot depicts the correlations between the predicted and estimated values across the years 2003–2019, using model 1 (E + A), 2 and 5, respectively.

Three cross-validation schemes and seven models were used for assessing the prediction performance for grain yield (kg/ha) using the drought stress and non-stress datasets. The three cross-validation scenarios imitate real prediction problems of interest for breeders, and hence would contribute in addressing the prediction problems that breeders might face at different stages of the breeding pipeline (Persa et al., 2021). In general, it was found that in the CV2 scenario, the predictive abilities were the highest when using both non-stress and drought stress data sets for model calibration. Models 6 (E + G1 + G2 + G12 + G1E + G2E + AE) and model 7 (E + GCA + A + GCA × E + A × E) performed superior to the other models in all the three considered datasets: stress, non-stress and combined data of drought stress and non-stress. Amongst the three scenarios, predictive abilities were poor in the CV0 scenario when predicting stress environments with non-stress datasets and vice versa. However, for CV1 and CV2 minimal differences in the predictive abilities were observed under this crossed validation. Overall, as expected the average correlation values for the scenario CV2 were highest followed by CV1 and CV0. Tables 1, 2 present the results for CV2 and CV1 schemes. Detailed results for three scenarios CV0, CV1, and CV2 with seven different models using the stress, non-stress and combined datasets are depicted in Figures 2–4, respectively.

**Table 1:** The predictive abilities for the three selection criterion’s; CV2, CV1 and CV0 analyzed to predict grain yields under non-stress conditions with calibration sets *viz.,* non-stress (NS) and Combined [All (stress and non-stress)] (CSN) datasets using seven models.

Mixed models	Cross-validation scenarios						
	CV2		CV1		CV0		
Calibration sets	CSN	NS	CSN	NS	CSN	NS	S → NS
M1: E + A	0.276	0.286	0.184	0.227	0.200	0.171	0.127
M2: E + A + AE	0.348	0.328	0.292	0.290	**0.209**	0.176	**0.132**
M3: E + GCA	0.272	0.304	0.204	0.247	0.159	0.153	0.095
M4: E + GCA + SCA	0.304	0.338	0.225	0.277	0.188	**0.193**	0.106
M5: E + GCA + SCA + GCA × E + SCA × E	0.352	0.347	**0.306**	0.305	0.197	0.190	0.112
M6: E + GCA + SCA + GCA × E + A × E	0.352	**0.348**	0.304	**0.309**	0.184	0.187	0.086
M7: E + GCA + A + GCA × E + A × E	**0.360**	0.341	0.298	0.299	0.206	0.182	0.126

Also the extreme right column of the table contains the predicted grain yield under non-stress conditions using the stress (S) dataset in CV0 scenario. The highlighted values represent models harboring highest predictive abilities in each of the cases.

**Table 2:** The predictive abilities for the three selection criterions; CV2, CV1 and CV0 analyzed to predict grain yields under stress (S) conditions using the calibration sets *viz.,* stress dataset alone and Combined [All (stress and non-stress)] (CSN) datasets using seven models.

Mixed models	Cross-validation scenarios						
	CV2		CV1		CV0		
Calibration sets	CSN	S	CSN	S	CSN	S	NS → S
M1: E + A	0.242	0.254	0.170	0.230	**0.185**	0.073	**0.171**
M2: E + A + AE	0.381	0.355	0.340	0.349	0.165	0.047	0.141
M3: E + GCA	0.254	0.281	0.197	0.256	0.157	0.060	0.140
M4: E + GCA + SCA	0.274	0.328	0.209	0.306	0.162	**0.125**	0.138
M5:E + GCA + SCA + GCA × E + SCA × E	0.374	**0.364**	0.345	**0.359**	0.146	0.108	0.122
M6: E + GCA + SCA + GCA × E + A × E	0.371	**0.364**	0.339	0.355	0.149	0.114	0.118
M7: E + GCA + A + GCA × E + A × E	**0.388**	0.362	**0.344**	0.355	0.176	0.073	0.149

Also, the extreme right column of the table contains the predicted grain yield under stress conditions using the non-stress (NS) dataset in CV0 scenario. The highlighted values represent models harboring highest predictive abilities in each of the cases.

### Prediction with cross-validation scenario CV2

The CV2 cross-validation scheme was created to assess the prediction performance of the incomplete trials. It was observed that the prediction performance of incomplete trials was marginally lower in predicting under non-stress conditions using the non-stress dataset (~0.348) compared to those obtained for predicting drought grain yields with the drought stress datasets (~0.354; Tables 1, 2; Figure 2) with the corresponding most successful model (M6). Similarly, the higher prediction accuracies for predicting grain yield occurred when combining data of stress and non-stress conditions (CNS) in calibration sets (~0.360 for NS and 0.388 for S). Overall, models 5 (E + GCA + SCA + GCA × E + SCA × E), model 6 (E + GCA + SCA + GCA × E + A × E) and model 7 (E + GCA + A + GCA × E + A × E) gave similar prediction accuracies when using data from both stress and non-stress datasets for model calibration. Most importantly, the inclusion of pedigree information and their interaction with the environment improved the prediction accuracy. This is evident from the prediction accuracies obtained for predicting the grain yields under stress and non-stress conditions using models 2 (0.328 and 0.381), model 5 (0.352 and 0.374), model 6 (0.352 and 0.371) and model 7 (0.360 and 0.388). Further, the models involving GCA and SCA effects and their interactions with the environment had a slight edge in improving the prediction performance over the basic interaction models, however, the pattern was not consistent across all the years. Furthermore, details of the non-stress and stress datasets for predicting each dataset and also their counterparts are elaborated below.

### Predictions (CV2) under non-stress conditions using data from non-stress and combined data sets

The non-stress and combined dataset was utilized to predict the genotype performances for grain yield under the non-stress conditions using seven models. The predictive abilities obtained for the non-stress conditions using the models 2 to 7 (0.328, 0.304, 0.338, 0.347, 0.348, 0.341) depicted a significant improvement in comparison to the conventional model 1 (E + A) (0.286), however the prediction abilities were minimally different amongst each other with model 6 (E + GCA + SCA + GCA × E + A × E) and model 5 (E + GCA + SCA + GCA × E + SCA × E) showing slightly higher predictive abilities of 0.348 and 0.347, respectively. The results were slightly different when predicting the grain yield using combined datasets, in which model 7 (E + GCA + A + GCA × E + A × E) depicted the highest predictive ability (0.360), with significant increase with respect to model 1 (E + A) and model 3 (E + GCA) with predictive abilities of 0.276 and 0.272; and comparable predictive abilities of 0.348, 0.304, 0.352, 0.352 obtained using the model 2 (E + A + A × E), model 4 (E + GCA + SCA), model 5 (E + GCA + SCA + GCA × E + SCA × E) and model 6 (E + GCA + SCA + GCA × E + A × E) respectively.

When we compare the average correlation values from predicting crop performance from years 2005 to 2019, the average correlation values for the years 2010 and 2016 were the highest amongst the others. The grain yield of the genotypes for the year 2016 when predicted using the cut-out years, model 2 (E + A + A × E) gave higher average correlation values of 0.55 when the calibration set was composed of the non-stress and combined datasets for predicting the non-stress conditions, followed by model 5 (E + GCA + SCA + GCA × E + SCA × E) and model 1 (E + A) with minor differences in the correlation values. Similarly, the average correlation values of the genotypes for the year 2010 using model 5 (E + GCA + SCA + GCA × E + SCA × E) gave higher average correlations of 0.55 (Figure 2A). Therefore, we can conclude that models 2 and 5 helped in predicting better results in comparison to the other five models.

### Predictions (CV2) under stress conditions using data from stress, and combined data sets

In predicting the performance of lines tested under stress conditions during some years but not in others, model 5 (E + GCA + SCA + GCA × E + SCA × E) and model 6 (E + GCA + SCA + GCA × E + A × E) performed equally superior over other models with predictive abilities of 0.364 each. However, when predicting the grain yield using the combined information of stress and non-stress environmental conditions for calibration, model 7 (E + GCA + A + GCA × E + A × E) performed better than other models with the predictive abilities of 0.388. Under this scenario, the conventional model 1 (E + A) gave the lowest predictive abilities of 0.242 and 0.254, followed by model 3 (E + GCA) with predictive abilities of 0.254 and 0.281, respectively. Overall, incorporating the pedigree and pedigree by environment interactions, genotype by genotype, genotype by environment interactions, along with the GCA and SCA components in the model 2 (E + A + A × E), model 4 (E + GCA + SCA), model 5 (E + GCA + SCA + GCA × E + SCA × E), model 6 (E + GCA + SCA + GCA × E + A × E) and model 7 (E + GCA + A + GCA × E + A × E) improved the predictive abilities significantly. Conversely when analyzed for predicting the grain yields combining stress and non-stress datasets as calibration sets the model 2 (E + A + A × E), model 5 (E + GCA + SCA + GCA × E + SCA × E), model 6 (E + GCA + SCA + GCA × E + A × E) and model 7 (E + GCA + A + GCA × E + A × E), harboring pedigree and pedigree by environment interactions; genotype and genotype by environment interactions along with GCA and SCA components gave improved predictive abilities of 0.381, 0.374, 0.371, 0.388, respectively.

When we compare the average correlation values from predictions of the genotypes for the year 2005 to 2019, the average correlation values for the years 2016 and 2010 were highest amongst the others (Figure 2B). The data for the genotypes of the year 2016 when predicted using the cut-out years, model 2 gave higher average correlation values of 0.55 with both non-stress and combined calibration sets, followed by Model 5 (E + GCA + SCA + GCA × E + SCA × E) and Model 1 (E + A) with minor differences in the correlation values. Similarly, the average correlation values of the genotypes for the year 2010 using Model 5 gave higher average correlations of 0.55 (Figure 2B).

### Prediction with cross-validation scenario CV1

In CV1 scenario lines that have been not evaluated are predicted. In predicting the grain yield of untested lines, model 5 (E + GCA + SCA + GCA × E + SCA × E) performed superiorly over the other models in all the three manners for composing training sets (stress, non-stress, and combined) for predicting trait performance of drought stress, and non-stress conditions (Tables 1, 2; Figure 3). Furthermore, how the CV1 scenario behaves while using the datasets of the non-stress and stress datasets for predicting each dataset and also their counterpart is given below.

### Predicting the performance under non-stress conditions using data (calibration) from non-stress and combined conditions

The highest prediction abilities using the non-stress and combined datasets for predicting the non-stress grain yields were obtained using model 6 (E + GCA + SCA + GCA × E + A × E) (0.309) and model 5 (E + GCA + SCA + GCA × E + SCA × E) (0.306) respectively. The minimal predictive abilities of 0.184 and 0.227 were found with the conventional model 1 (E + A) alike in the CV2 scenario. Overall, the inclusion of genotype by environmental interaction effects along with the GCA and SCA by environment components in the model 5 (E + GCA + SCA + GCA × E + SCA × E) and genotype by environment interactions with the pedigree by environment interactions in the model 6 (E + GCA + SCA + GCA × E + A × E) improved the predictive abilities with both non-stress (0.305; 0.309) and combined calibration sets (0.306; 0.304) for predicting non-stress conditions. However unlike the CV2 scenario [where, predictive abilities were 0.341 (non-stress dataset) and 0.360 (combined dataset)], model 7 (E + GCA + A + GCA × E + A × E) depicted comparatively lower predictive ability of 0.298 and 0.299 using non-stress and combined datasets to model 5 (E + GCA + SCA + GCA × E + SCA × E) and 6 (E + GCA + SCA + GCA × E + A × E) with predictive abilities of 0.305 and 0.309 (non-stress dataset); 0.306 and 0.304 (combined dataset), respectively. The average correlation values were obtained from predictions of the genotypes for the year 2005 to 2019. The average correlation values for the years 2010 were highest followed by 2012, 2013, 2016 and 2018 when predicting the non-stress conditions with average correlation values of 0.58, 0.48, 0.48, 0.47, 0.47, respectively. In all of the years, except 2005, 2009 and 2019 model 5 (E + GCA + SCA + GCA × E + SCA × E) gave higher average correlation values when the calibration set was composed of the combined datasets (NS and S; Figure 3A).

### Predictive abilities under stress conditions using data (calibration) from stress and combined conditions

The prediction abilities for predicting under stress conditions using data from the stress (historical reproductive stage drought stress dataset) and combined conditions for model calibration were similar to those obtained under the CV2 scenario. When conducting the model calibration using data from the stress conditions, the predictive abilities ranged between 0.230 using model 1 (E + A) to 0.359 with model 5 (E + GCA + SCA + GCA × E + SCA × E). The added interaction effects including the genotype by genotype, genotype by environment, SCA by environment and GCA by environment interactions in model 5 (E + GCA + SCA + GCA × E + SCA × E) gave significantly higher predictive abilities of 0.359 in comparison to the conventional model 1 (E + A), model 3 (E + GCA) and model 4 (E + GCA + SCA) with environment, GCA and SCA interactions giving prediction abilities of 0.230, 0.256 and 0.306, respectively. However, there were minimal differences in predictive abilities using model 5 (E + GCA + SCA + GCA × E + SCA × E) (0.359) compared with model 6 (E + GCA + SCA + GCA × E + A × E) (0.355) and model 7 (E + GCA + A + GCA × E + A × E) (0.355), respectively. This signifies the added environment interactions effects of genotype by environment, pedigree by environment, GCA by environment and SCA by environment interactions contributed to improving the predictive abilities when predicting stress environments using stress calibration sets.

Also, when using the combined calibration set for predicting the stress conditions, model 5 (E + GCA + SCA + GCA × E + SCA × E) gave the highest predictive abilities of 0.345 alike as obtained using the stress dataset with slight differences with model 2 (E + A + A × E) (0.340), model 6 (E + GCA + SCA + GCA × E + A × E) (0.339) and model 7 (E + GCA + A + GCA × E + A × E) (0.344), respectively. Likewise, model 1 (E + A), model 3 (E + GCA) and model 4 (E + GCA + SCA) gave significantly lower prediction abilities of 0.170, 0.197 and 0.209.

When observed across years/environments, the average correlation values of the genotypes with the stress dataset for predicting the grain yields under the stress conditions for the years 2003–2019, depicted highest values for the year 2010 with the average correlation values of 0.60 using the model 5 (E + GCA + SCA + GCA × E + SCA × E). This was followed by the years 2012 and 2003 with average correlation values of 0.58 and 0.48 using the combined datasets using the model 5 (E + GCA + SCA + GCA × E + SCA × E) and model 2 (E + A + A × E), respectively. The average correlations for the years 2004, were minimal amongst all followed by 2009, 2005, 2017 and 2019. Overall, using model 5 (E + GCA + SCA + GCA × E + SCA × E) and model 2 (E + A + A × E) average correlation values were higher over the conventional model 1 (E + A) (Figure 3B).

### Predicting the performance for CV0 scenario

In predicting the performance in new environment/year (forward prediction/leave-one-year-out) under scenario CV0, model 4 (E + GCA + SCA) including the genotypes by genotype interactions gave higher predictive abilities under both stress and non-stress conditions showing predictive abilities of 0.125 and 0.193 under reproductive stage drought stress and non-stress conditions, respectively. However, when using the combined dataset for calibration, model 2 (E + A + A × E) gave highest prediction accuracies of 0.209 compared to stress and non-stress counterparts. The CV0 scenario was peculiar than the other two scenarios as it was designed not only to predict the grain yields under stress and non-stress conditions with the respective datasets but also the counterpart datasets were used to predict each condition (i.e., stress to predict non-stress conditions and *vice-versa*). Overall, amongst the three tested calibration sets of non-stress, combined and counterpart predictions, combined dataset gave highest prediction accuracies of 0.209, 0.206 and 0.200 using model 2, 7 and 1, respectively.

### Predictive abilities under non-stress using data from non-stress and combined conditions for model calibration

The predictive abilities of all the seven models for predicting the genotype performance in new environments were nearly similar with minor differences; however, were unlike the results obtained in the other scenarios CV1 and CV2. The best results were obtained from implementing model 4 (E + GCA + SCA) with estimated predictive ability of 0.193 using the non-stress dataset. The predictive abilities using the combined dataset for model training were comparable when using model 2 (E + A + A × E) and model 7 (E + GCA + SCA + GCA × E + A × E), however model 2 (E + A + A × E) gave higher predictive ability of 0.209. The model 3 (E + GCA), unlike other two scenarios and conditions, showed the least predictive abilities of 0.153 and 0.159 using both the non-stress and combined calibration sets. Additionally, for specially designed case for predicting the grain yields under non-stress conditions using the stress dataset, model 2 (E + A + A × E) gave a maximum prediction accuracy of 0.132. Also, models 1 and 7 gave comparable results with predictive abilities of 0.127 and 0.126, respectively.

In the year wise comparison, an average correlation value of 0.5 was observed in the year 2016, followed by the years 2015, 2003 and 2007 amongst the 15 years from 2005 to 2019. Model 5 performed superior over the other models 1 and 2 when analyzed using the non-stress as well as combined datasets. Also, there were minimal differences between the three plotted models for the year 2016 (Figure 4A).

### Predictive abilities under stress using data from stress and combined conditions for model calibration

The predictive abilities for the scenario CV0 for predicting the new environments using the stress and combined datasets for model training were almost alike the results obtained for the non-stress dataset. The inclusion of the models with pedigree effects improved the predictive abilities. The model 4 (E + GCA + SCA) gave improved predictive abilities of 0.125 using the stress dataset like with the non-stress dataset. Unlike the results obtained for the other two scenarios and conditions, model 1 (E + A) performed better than other models in predicting yield when combined calibration conditions were used for training with the predictive ability of 0.185, with minimal differences to model 7 (E + GCA + A + GCA × E + A × E) (0.176). Congruently, in the case to predict the grain yields using the non-stress dataset for predicting the stress environments, model 1 again gave maximum predictive ability (0.171) followed by model 7 (0.149).

In the comparative analysis amongst the tested years or environments, an average correlation value of 0.5 was observed in the year 2016, followed by the years 2015, 2003 and 2007 amongst the 15 years from 2005 to 2019. Model 5 performed superior over the other models 1 and 2 when using the non-stress as well as combined datasets for model calibration. Also, there were minimal differences between the three plotted models for the year 2016 (Figure 3B).

## Discussion

In multi-environment trials, G × E prediction models for complex traits like grain yield could help in accelerating the breeding cycles. Integration of G × E in predictions has helped to improve the genetic gains significantly (Crespo-Herrera et al., 2017; Jarquín et al., 2017; Bajgain et al., 2020; Dreisigacker et al., 2021). Models based on G × E accounting for the correlated environmental effects might significantly help to enhance the predictive ability of un-observed phenotypes in multi-environment trials (Sukumaran et al., 2017). Here, we leverage the huge number of multi-year environmental data along with robust pedigree information to exploit the genetic variance and covariance structure of correlated environmental effects to predict new lines or environments, and dissect the G × E by considering the interaction of the GCA and SCA terms with environment and these were modeled using the pedigree-relationship matrix.

### Pedigree information to accesses predictions

The availability of robust pedigree information was pivotal in this study, which helped us fit the additive matrix in the second stage analysis to estimate breeding values and prediction accuracies (Khanna et al., 2022). The essence of using the pedigree-based kinship matrix is that it accounts for the additive genetic co-variance between the genotypes to estimate the breeding values (Piepho et al., 2008). Robust and deep pedigrees may result in the expected genetic relationships close to realized estimated relationships obtained by genetic markers (Howard et al., 2019). Further, it has been shown that with robust and deep pedigree records, pedigree-based models perform much better than genetic marker-based models (Juliana et al., 2017). For example, Howard et al. (2019) showed that pedigree-based models gave better prediction accuracy than genome-based models when main effects were modeled. However, we emphasize that the robust pedigree information might be incorporated with the genetic marker-based information to improve the accuracy of prediction models to a greater extent.

### Identify best model and integrate interaction and genotypic effects (GCA and SCA)

The seven models ranging from basic models without any interaction effects to higher advanced models involving the interaction and main genotypic effects were utilized to assess the predictive ability. In general, the incorporation of genotype-by-environment interactions (pedigree-by-environment; A × E here) in prediction models increased the predictive ability in all three scenarios. Dissecting and quantifying A × E has been an integral part of predictions and has led to increased prediction ability (Jarquín et al., 2017; Ankamah-Yeboah et al., 2020), as found in this study. Further models, involving the GCA and SCA effects along with the A × E interactions have slightly better predictive ability as compared to those only involving the GCA and SCA effects. This gain is not surprising and may be due to the significant contribution of variance components contributed by the interaction effects. Thus, we argue that reasonable gains in the prediction ability can be achieved by jointly considering the main and environmental interaction components as depicted in previous studies on wheat (Burgueño et al., 2012; Sukumaran et al., 2017) and barley (Ankamah-Yeboah et al., 2020).

As expected, under the CV0 scenario, models accounting for the A × E interactions have very low predictive ability since no phenotypic information from the target environment is available. CV0, which involves the prediction performance of the genotypes in the new and un-observed environments, typically fails to exploit the genetic correlation among the different environments (Jighly et al., 2021). This results in lower prediction accuracies. Similar results of lower accuracy under the CV0 scenario have been observed in wheat and Barley Howard et al. (2019); (Ankamah-Yeboah et al., 2020). To predict the new environments and dissect G × E, it is important to exploit and use the genetic correlations among different sets of environments for improved and accurate estimates of genomic estimated breeding values (GEBVs). Predicting the new and unobserved environments without any information on genetic correlations among the environments or without borrowing information from other environments would be challenging, resulting in lower accuracy of breeding values. Recently, Jighly et al. (2021) proposed a novel alternative strategy called 3GS that predicts phenotypes in unobserved environments with high prediction accuracy with low to negative correlations among the different environments.

### Predictions under different scenarios

Importantly, the prediction abilities were majorly dependent on the employed cross-validation schemes used in this study. In general, prediction ability was marginally lower when predicting non-stress lines using stress data and *vice-versa*. Much better prediction accuracies were obtained when predicting the lines using the data sets evaluated under the same conditions (stress or non-stress data sets). This may be expected due to extremely different experimental conditions under drought, and normal conditions resulting in lower correlations and lower heritability always observed under the drought conditions. Thus, the alternative strategy must be used to predict the lines using data from different conditions (Lyra et al., 2017). For example, here in this study, when we combine both stress and non-stress for model calibration, we observed much higher prediction accuracies under all the three prediction scenarios.

Further, amongst the three scenarios, CV1 and CV2 showed higher prediction accuracies as compared to the CV0. As discussed above, CV0 in which un-observed environments are predicted is not a good option to accurately predict as they do not borrow information from other related environments; however, this an important prediction scenario in breeding programs. In comparison to CV0, CV1, and CV2 scenarios in which new lines are tested or predicted borrows information from other and same environments (years). Thus, CV2 and CV1 scenarios, allows to exploit the genetic correlations amongst different environments and thus impart in improving the prediction accuracies (Ben Hassen et al., 2018; Ankamah-Yeboah et al., 2020). Notably, in predicting the performance of new lines under CV1, the combined effects of GCA, SCA, A × E interaction components contributed to improve the predictive ability. Hence, adding additive and A × E interaction components could contribute in enhancing the prediction abilities for deducing grain yield performances of incomplete trials (CV2).

## Conclusion

Historical data sets have been a great resource to deduce meaningful conclusions for the implementation and optimization of pedigree-based predictions in breeding programs. Here, we leveraged a relatively large environmental dataset to answer the key questions related to predictions in the IRRI’s rice drought breeding program. We proposed a series of models that were compared with conventional pedigree-based implementation for a total of 7 models that were tested under three validation schemes to dissect the G × E. We found that prediction accuracies improved when incorporating the interaction components helping to build the future pedigree or genomic-based prediction models in the rice breeding program. Prediction accuracy varies considerably across the validation schemes, with CV0 as expected, giving the minimal prediction accuracy, highlighting the importance of borrowing the correlated information from other environments to improve prediction accuracy. Further, we emphasize the inclusion of a marker-based relationship matrix will further help in improving the accuracy of genomic prediction models and answer the question in a more meaningful way. Conclusively, rigorous theoretical and empirical assessment of genomic prediction optimization approaches will be crucial in translating the investments into real genetic gains in rice breeding programs.

## Data availability statement

The original contributions presented in the study are included in the article/supplementary material, further inquiries can be directed to the corresponding authors.

## Author contributions

WH, SB, and DJ designed the concept and study. WH, DJ, and AK draw the initial draft of the manuscript and performed the prediction analysis and phenotypic data analysis. MC and MA helped in data collection and formatting the manuscript. All authors contributed to the article and approved the submitted version.

## Funding

The study was funded by AGGRi Alliance project, “Accelerated Genetic Gains in Rice Alliance” Grant ID: OPP1194925- INV 008226, funded by the Bill and Melinda Gates Foundation.

## Conflict of interest

The authors declare that the research review was conducted in the absence of any commercial or economic associations that could be construed as a potential conflict of interest.

## Publisher’s note

All claims expressed in this article are solely those of the authors and do not necessarily represent those of their affiliated organizations, or those of the publisher, the editors and the reviewers. Any product that may be evaluated in this article, or claim that may be made by its manufacturer, is not guaranteed or endorsed by the publisher.

## References

[ref1] Albrecht T. Wimmer V. Auinger H.-J. Erbe M. Knaak C. Ouzunova M.. (2011). Genome-based prediction of testcross values in maize. Theor. Appl. Genet. 123, 339–350. doi: 10.1007/s00122-011-1587-7 21505832

[ref2] Amadeu R. R. Cellon C. Olmstead J. W. Gracia A. A. Resende M. F. Muñoz P. R. (2016). AGHmatrix: R package to construct relationship matrices for autotetraploid and diploid species: a blueberry example. Plant Genome 9:3. doi: 10.3835/plantgenome2016.01.0009 27902800

[ref3] Ankamah-Yeboah T. Janss L. L. Jensen J. D. Hjortshøj R. L. Rasmussen S. K. (2020). Genomic selection using pedigree and marker-by-environment interaction for barley seed quality traits from two commercial breeding programs. Front. Plant Sci. 11:539. doi: 10.3389/fpls.2020.00539 32457780 PMC7227446

[ref4] Bajgain P. Zhang X. Anderson J. A. (2020). Dominance and G×E interaction effects improve genomic prediction and genetic gain in intermediate wheatgrass (*Thinopyrum intermedium*). Plant Genome 13:e20012. doi: 10.1002/tpg2.20012 33016625 PMC12806932

[ref5] Ben Hassen M. Bartholomé J. Valè G. Cao T. V. Ahmadi N. (2018). Genomic prediction accounting for genotype by environment interaction offers an effective framework for breeding simultaneously for adaptation to an abiotic stress and performance under normal cropping conditions in rice. G3 (Bethesda) 8, 2319–2332. doi: 10.1534/g3.118.200098 29743189 PMC6027893

[ref6] Bernal-Vasquez A.-M. Utz H.-F. Piepho H.-P. (2016). Outlier detection methods for generalized lattices: a case study on the transition from ANOVA to REML. Theor. Appl. Genet. 129, 787–804. doi: 10.1007/s00122-016-2666-6 26883044

[ref8] Burgueño J. de los Campos G. Weigel K. Crossa J. (2012). Genomic prediction of breeding values when modeling genotype× environment interaction using pedigree and dense molecular markers. Crop Sci. 52, 707–719. doi: 10.2135/cropsci2011.06.0299

[ref9] Collard B. C. Y. Gregorio G. B. Thomson M. J. Islam M. R. Vergara G. V. Laborte A. G.. (2019). Transforming Rice breeding: re-designing the irrigated breeding pipeline at the international Rice research institute (IRRI). Crop Breed. Genet. Genom. 1:e190008. doi: 10.20900/cbgg20190008

[ref10] Crespo-Herrera L. A. Crossa J. Huerta-Espino J. Autrique E. Mondal S. Velu G.. (2017). Genetic yield gains in CIMMYT’s international elite spring wheat yield trials by modeling the genotype × environment interaction. Crop Sci. 57, 789–801. doi: 10.2135/cropsci2016.06.0553 33343008 PMC7680939

[ref11] Crossa J. de los Campos G. Pérez P. Gianola D. Burgueño J. Araus J. L.. (2010). Prediction of genetic values of quantitative traits in plant breeding using pedigree and molecular markers. Genetics 186, 713–724. doi: 10.1534/genetics.110.118521 20813882 PMC2954475

[ref12] Cullis B. R. Smith A. B. Coombes N. E. (2006). On the design of early generation variety trials with correlated data. JABES 11, 381–393. doi: 10.1198/108571106X154443

[ref13] Damesa T. M. Möhring J. Worku M. Piepho H. P. (2017). One step at a time: stage-wise analysis of a series of experiments. Agronomy 109, 845–857. doi: 10.2134/agronj2016.07.0395

[ref14] Dawson J. C. Endelman J. B. Heslot N. Crossa J. Poland J. Dreisigacker S.. (2013). The use of unbalanced historical data for genomic selection in an international wheat breeding program. Field Crops Res. 154, 12–22. doi: 10.1016/j.fcr.2013.07.020

[ref15] Dreisigacker S. Crossa J. Pérez-Rodríguez P. Montesinos-López O. A. Rosyara U. Juliana P.. (2021). Implementation of genomic selection in the CIMMYT global wheat program, findings from the past 10 years. Crop Breed. Genet. Genom. 3:e210005. doi: 10.20900/cbgg20210005

[ref16] Henry T. Nguyen R. Chandra B. Blum A. (1997). Breeding for drought resistance in Rice: physiology and molecular genetics considerations. Crop Sci. 37, 1426–1434. doi: 10.2135/cropsci1997.0011183X003700050002x

[ref17] Howard R. Gianola D. Montesinos-López O. Juliana P. Singh R. Poland J.. (2019). Joint use of genome, pedigree, and their interaction with environment for predicting the performance of wheat lines in new environments. G3 (Bethesda) 9, 2925–2934. doi: 10.1534/g3.119.400508 31300481 PMC6723131

[ref18] Hunt C. H. Van Eeuwijk F. A. Mace E. S. Hayes B. J. Jordan D. R. (2018). Development of genomic prediction in sorghum. Crop Sci. 58, 690–700. doi: 10.2135/cropsci2017.08.0469

[ref19] Hussain W. Anumalla M. Catolos M. Khanna A. Sta. Cruz M. T. Ramos J.. (2022). Open-source analytical pipeline for robust data analysis, visualizations and sharing in crop breeding. Plant Methods 18:14. doi: 10.1186/s13007-022-00845-7 35123539 PMC8817612

[ref20] Jarquín D. Crossa J. Lacaze X. Du Cheyron P. Daucourt J. Lorgeou J.. (2014). A reaction norm model for genomic selection using high-dimensional genomic and environmental data. Theor. Appl. Genet. 127, 595–607. doi: 10.1007/s00122-013-2243-1 24337101 PMC3931944

[ref21] Jarquin D. de Leon N. Romay C. Bohn M. Buckler E. S. Ciampitti I.. (2021). Utility of climatic information via combining ability models to improve genomic prediction for yield within the genomes to fields maize project. Front. Genet. 11:592769. doi: 10.3389/fgene.2020.592769 33763106 PMC7982677

[ref22] Jarquín D. Lemes da Silva C. Gaynor R. C. Poland J. Fritz A. Howard R.. (2017). Increasing genomic-enabled prediction accuracy by modeling genotype × environment interactions in Kansas wheat. Plant. Genome 10:1. doi: 10.3835/plantgenome2016.12.0130 28724062

[ref23] Jighly A. Hayden M. Daetwyler H. (2021). Integrating genomic selection with a genotype plus genotype x environment (GGE) model improves prediction accuracy and computational efficiency. Plant Cell Environ. 44, 3459–3470. doi: 10.1111/pce.14145 34231236

[ref24] Juliana P. Singh R. P. Singh P. K. Crossa J. Huerta-Espino J. Lan C.. (2017). Genomic and pedigree-based prediction for leaf, stem, and stripe rust resistance in wheat. Theor. Appl. Genet. 130, 1415–1430. doi: 10.1007/s00122-017-2897-1 28393303 PMC5487692

[ref25] Juma R. U. Bartholomé J. Thathapalli Prakash P. Hussain W. Platten J. D. Lopena V.. (2021). Identification of an elite Core panel as a key breeding resource to accelerate the rate of genetic improvement for irrigated Rice. Rice 14:92. doi: 10.1186/s12284-021-00533-5 34773509 PMC8590642

[ref26] Khanna A. Anumalla M. Catolos M. Bartholomé J. Fritsche-Neto R. Platten J. D.. (2022). Genetic trends estimation in IRRIs Rice drought breeding program and identification of high yielding drought-tolerant lines. Rice 15:14. doi: 10.1186/s12284-022-00559-3 35247120 PMC8898209

[ref27] Krishnappa G. Savadi S. Tyagi B. S. Singh S. K. Mamrutha H. M. Kumar S.. (2021). Integrated genomic selection for rapid improvement of crops. Genomics 113, 1070–1086. doi: 10.1016/j.ygeno.2021.02.007 33610797

[ref28] Kumar A. Raman A. Yadav S. Verulkar S. B. Mandal N. P. Singh O. N.. (2021). Genetic gain for rice yield in rainfed environments in India. Field Crops Res. 260:107977. doi: 10.1016/j.fcr.2020.107977 33390645 PMC7722510

[ref29] Kumar R. Venuprasad R. Atlin G. N. (2007). Genetic analysis of rainfed lowland rice drought tolerance under naturally-occurring stress in eastern India: heritability and QTL effects. Field Crops Res. 103, 42–52. doi: 10.1016/j.fcr.2007.04.013

[ref30] Lado B. Barrios P. G. Quincke M. Silva P. Gutiérrez L. (2016). Modeling genotype × environment interaction for genomic selection with unbalanced data from a wheat breeding program. Crop Sci. 56, 2165–2179. doi: 10.2135/cropsci2015.04.0207

[ref31] Li H. Rasheed A. Hickey L. T. He Z. (2018). Fast-forwarding genetic gain. Trends Plant Sci. 23, 184–186. doi: 10.1016/j.tplants.2018.01.007 29426713

[ref32] Lyra D. H. de Freitas Mendonça L. Galli G. Alves F. C. Granato Í. S. C. Fritsche-Neto R. (2017). Multi-trait genomic prediction for nitrogen response indices in tropical maize hybrids. Mol. Breed. 37, 1–14. doi: 10.1007/s11032-017-0681-128127252

[ref33] McLaren C. G. Bruskiewich R. M. Portugal A. M. Cosico A. B. (2005). The international Rice information system. A platform for meta-analysis of rice crop data. Plant Physiol. 139, 637–642. doi: 10.1104/pp.105.063438 16219924 PMC1255983

[ref34] Meuwissen T. H. Hayes B. J. Goddard M. E. (2001). Prediction of total genetic value using genome-wide dense marker maps. Genetics 157, 1819–1829. doi: 10.1093/genetics/157.4.1819 11290733 PMC1461589

[ref35] Ovenden B. Milgate A. Wade L. J. Rebetzke G. J. Holland J. B. (2018). Accounting for genotype-by-environment interactions and residual genetic variation in genomic selection for water-soluble carbohydrate concentration in wheat. G3 (Bethesda) 8, 1909–1919. doi: 10.1534/g3.118.200038 29661842 PMC5982820

[ref36] Peng S. Huang J. Sheehy J. E. Laza R. C. Romeo M. Visperas Zhong X.. (2004). Rice yields decline with higher night temperature from global warming. PNAS 101, 9971–9975. doi: 10.1073/pnas.0403720101 15226500 PMC454199

[ref37] Pérez-Rodríguez P. Crossa J. Bondalapati K. De Meyer G. Pita F. de los Campos G. (2015). A pedigree-based reaction norm model for prediction of cotton yield in multienvironment trials. Crop Sci. 55, 1143–1151. doi: 10.2135/cropsci2014.08.0577

[ref38] Persa R. Grondona M. Jarquin D. (2021). Development of a genomic prediction pipeline for maintaining comparable sample sizes in training and testing sets across prediction schemes accounting for the genotype-by-environment interaction. Agriculture 11:932. doi: 10.3390/agriculture11100932

[ref39] Philipp N. Weise S. Oppermann M. Börner A. Keilwagen J. Kilian B.. (2019). Historical phenotypic data from seven decades of seed regeneration in a wheat ex situ collection. Sci. Data 6:137. doi: 10.1038/s41597-019-0146-y 31358775 PMC6662709

[ref40] Piepho H. P. Möhring J. (2007). Computing heritability and selection response from unbalanced plant breeding trials. Genetics 177, 1881–1888. doi: 10.1534/genetics.107.074229 18039886 PMC2147938

[ref41] Piepho H. P. Möhring J. Melchinger A. E. Büchse A. (2008). BLUP for phenotypic selection in plant breeding and variety testing. Euphytica 161, 209–228. doi: 10.1007/s10681-007-9449-8

[ref42] Piepho H.-P. Möhring J. Schulz-Streeck T. Ogutu J. O. (2012). A stage-wise approach for the analysis of multi-environment trials. Biom. J. 54, 844–860. doi: 10.1002/bimj.201100219 23007738

[ref43] Rogers A. Dunne J. Romay M. Bohn M. Buckler E. Ciampitti I.. (2021). The importance of dominance and genotype-by-environment interactions on grain yield variation in a large-scale public cooperative maize experiment. G3-Genes Genom. Genet. 11:jkaa050. doi: 10.1093/g3journal/jkaa050 PMC8022981 33585867

[ref44] Rogers A. R. Holland J. B. (2022). Environment-specific genomic prediction ability in maize using environmental covariates depends on environmental similarity to training data. G3 (Bethesda) 12:jkab440. doi: 10.1093/g3journal/jkab440 35100364 PMC9245610

[ref45] Rutkoski J. Poland J. Mondal S. Autrique E. Pérez L. G. Crossa J.. (2016). Canopy temperature and vegetation indices from high-throughput phenotyping improve accuracy of pedigree and genomic selection for grain yield in wheat. G3 (Bethesda) 6, 2799–2808. doi: 10.1534/g3.116.032888 27402362 PMC5015937

[ref46] Smith A. B. Cullis B. R. (2018). Plant breeding selection tools built on factor analytic mixed models for multi-environment trial data. Euphytica 214:143. doi: 10.1007/s10681-018-2220-5

[ref47] Sukumaran S. Crossa J. Jarquin D. Lopes M. Reynolds M. P. (2017). Genomic prediction with pedigree and genotype× environment interaction in spring wheat grown in south and West Asia, North Africa, and Mexico. G3 (Bethesda) 7, 481–495. doi: 10.1534/g3.116.036251 27903632 PMC5295595

[ref48] Velazco J. G. Malosetti M. Hunt C. H. Mace E. S. Jordan D. R. Van Eeuwijk F. A. (2019). Combining pedigree and genomic information to improve prediction quality: an example in sorghum. Theor. Appl. Genet. 132, 2055–2067. doi: 10.1007/s00122-019-03337-w 30968160 PMC6588709

[ref49] Xu L. Yuan S. Wang X. Yu X. Peng S. (2021). High yields of hybrid rice do not require more nitrogen fertilizer than inbred rice: a meta-analysis. Food Energy Secur. 10, 341–350. doi: 10.1002/fes3.276

